# Supplementary material for: High Expression of ENO1 and Low Levels of Circulating Anti-ENO1 Autoantibodies in Patients with Myelodysplastic Neoplasms and Acute Myeloid Leukaemia
Source: Cancers (Basel). 2024 Feb 22;16(5):884. doi: 10.3390/cancers16050884 (PMC10931177; doi:10.3390/cancers16050884)

Supplemental Figure S1: Kaplan Meier plots and associated data used for meta-analysis of ENO1 mRNA expression with overall survival in AML. Datasets queried using (A) cBioPortal, (B) PrognoScan and (C) PRECOG

A

cBioPortal

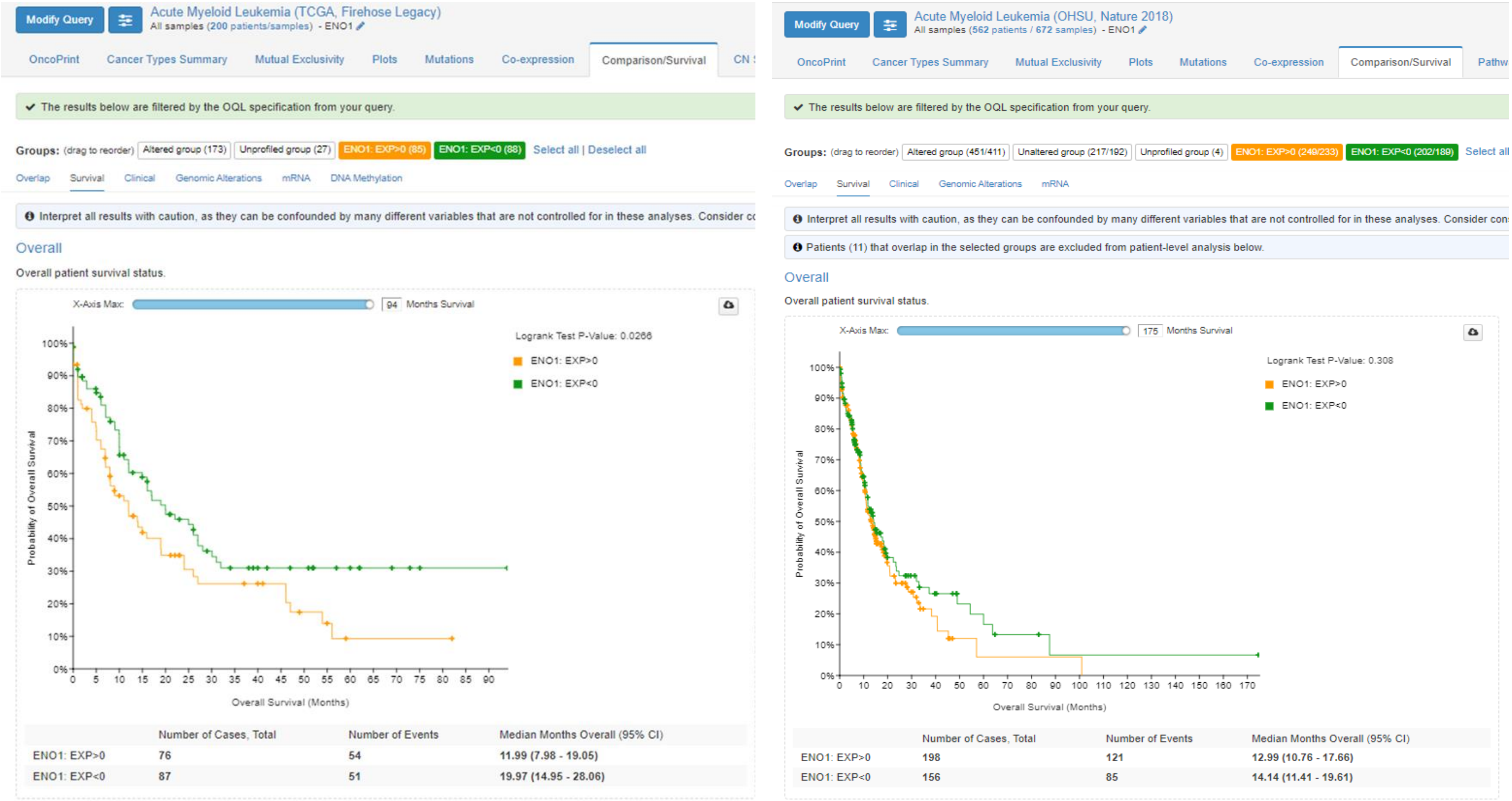

B

PrognoScan

DATA POSTPROCESSING	None
PROBE_NAME	201231_s_at [HG-U133A]
PROBE_DESCRIPTION	Homo sapiens enolase 1, (alpha) (ENO1), mRNA
GENE_SYMBOL	ENO1
GENE_DESCRIPTION	enolase 1, (alpha)
DATASET	GSE5122
CANCER_TYPE	Blood cancer
SUBTYPE	AML
N	58
ENDPOINT	Overall Survival
PERIOD	Days
COHORT	San Diego
ARRAY TYPE	HG-U133A
CONTRIBUTOR	Raponi
DATA_PROCESSING	MAS5
CUTPOINT	0.90
MINIMUM P-VALUE	0.001785
CORRECTED P-VALUE	0.041245
$\ln(HR_{high} / HR_{low})$	1.36
COX P-VALUE	0.129964
ln(HR)	0.35
HR [95% CI]	1.42 [0.90 - 2.23]

DATA POSTPROCESSING	None
PROBE_NAME	201231_s_at [HG-U133A]
PROBE_DESCRIPTION	Homo sapiens enolase 1, (alpha) (ENO1), mRNA
GENE_SYMBOL	ENO1
GENE_DESCRIPTION	enolase 1, (alpha)
DATASET	GSE8970
CANCER_TYPE	Blood cancer
SUBTYPE	AML
N	34
ENDPOINT	Overall Survival
PERIOD	Days
COHORT	San Diego
ARRAY TYPE	HG-U133A
CONTRIBUTOR	Raponi
AGE	63-85
DATA_PROCESSING	MAS5
POST-TREATMENT	Tipifarnib: 100%
PRE-TREATMENT	NONE
CUTPOINT	0.62
MINIMUM P-VALUE	0.197725
CORRECTED P-VALUE	-
$\ln(HR_{high} / HR_{low})$	0.51
COX P-VALUE	0.218778
ln(HR)	0.29
HR [95% CI]	1.33 [0.84 - 2.12]

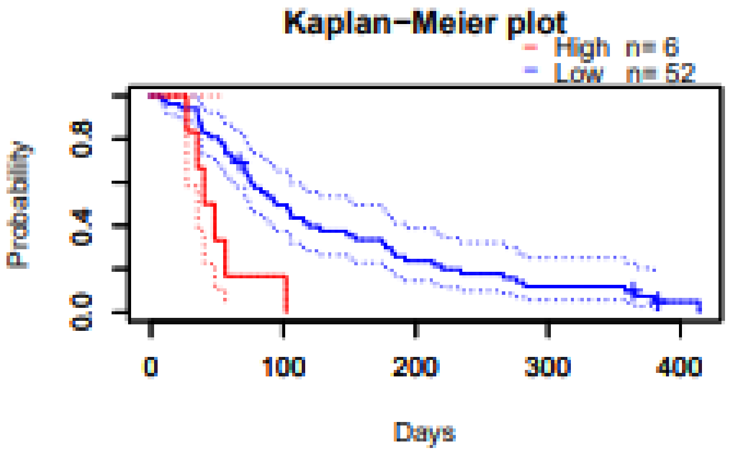

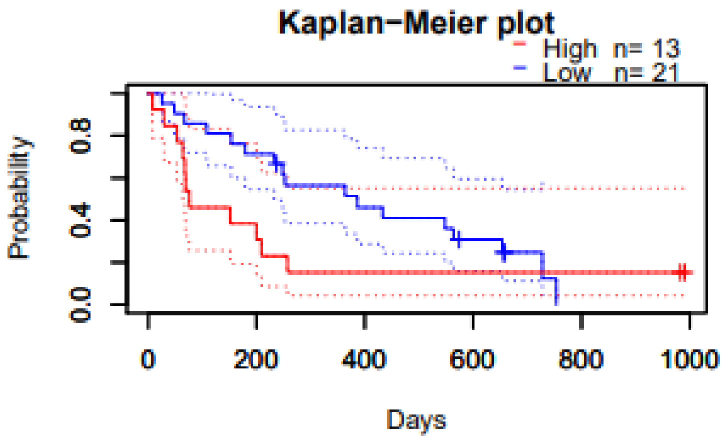

C

PRECOG

Accession: GSE12417
Author: Metzeler
Number of patients (OS/DSS): 405

2023_at ENO1 - enolase 1, (alpha)

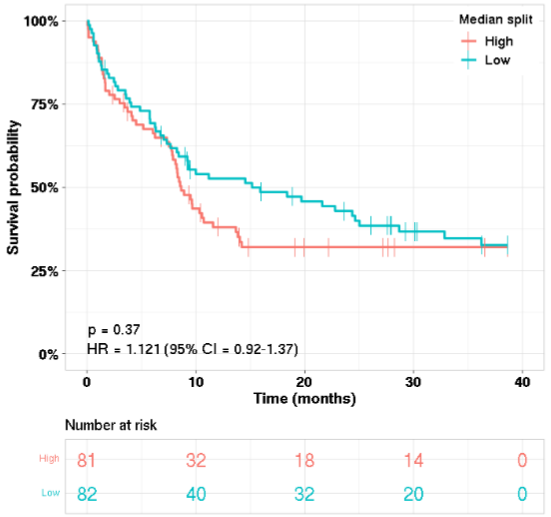

Accession: GSE12417
Author: Metzeler
Number of patients (OS/DSS): 405

2023_at ENO1 - enolase 1, (alpha)

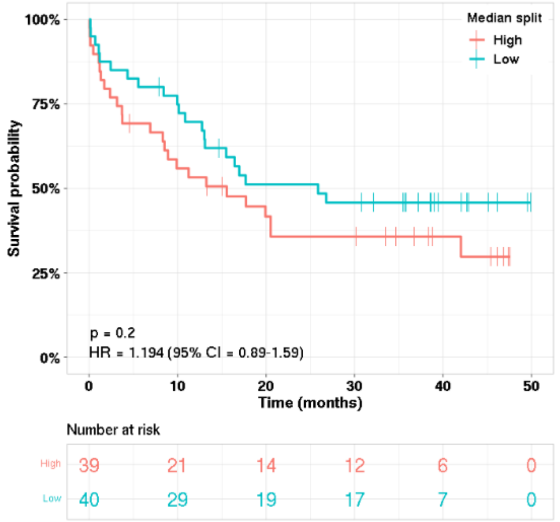

PubMed: Yamashita et al. (opens in new browser window/tab)
Accession: GSE1427 (opens in new browser window/tab)
No. patients (OS/DSS): 137

201231_s_at - ENO1 - enolase 1, (alpha)

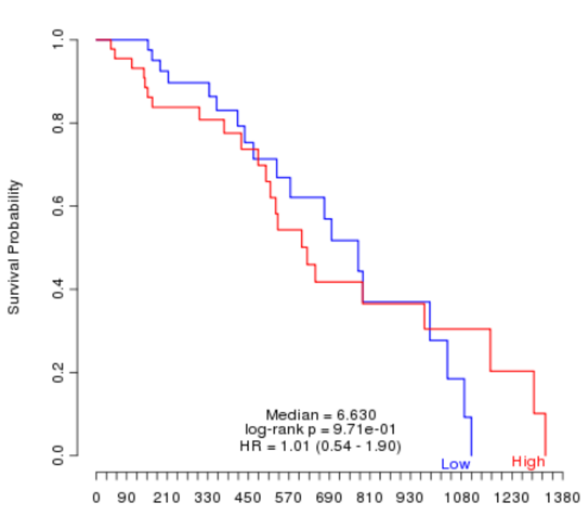

Accession: ca00119
Author: Wilson
Number of patients (OS/DSS): 170

2023_at ENO1 - enolase 1, (alpha)

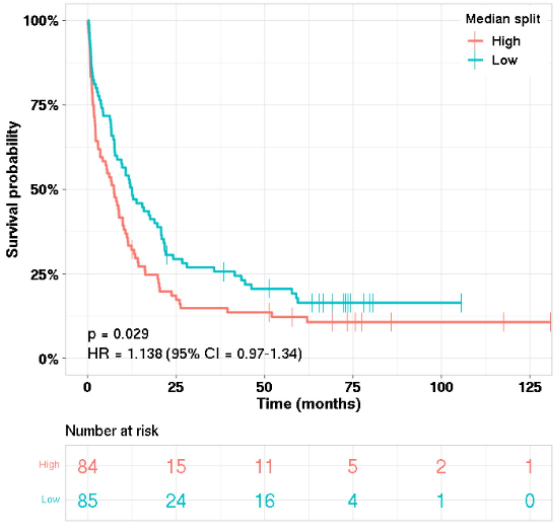

Accession: GSE14468
Author: Wouters
Number of patients (OS/DSS): 262

2023_at ENO1 - enolase 1, (alpha)

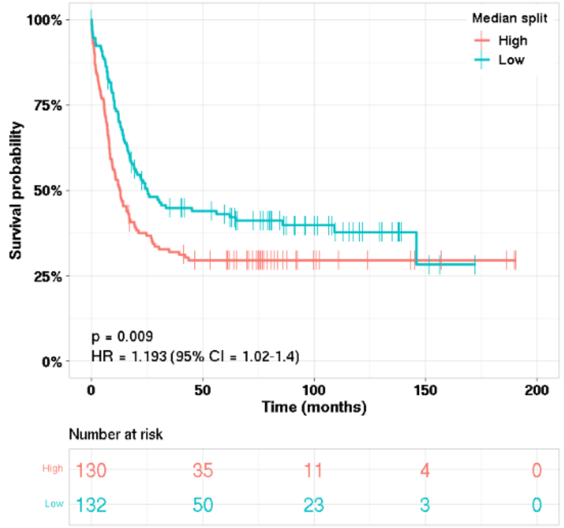

Supplement: Supplementary file 1 [file cancers-16-00884-s001.zip › cancers-2808695-supplementary.pdf]
